# Zeolite-supplemented diets in the prenatal period affected postpartum reproductive parameters, colostrum production, and body condition score of dairy cows

**DOI:** 10.5455/javar.2023.j694

**Published:** 2023-09-24

**Authors:** Nader Salman Movahedi, Farhad Foroudi, Naser Karimi, Mohammad Reza Abedini, Kazem Karimi

**Affiliations:** Department of Animal Science, Varamin-Pishva Branch, Islamic Azad University, Varamin, Iran

**Keywords:** BCS, colostrum, dairy cows, hypocalcemia, reproductive disorder, zeolite

## Abstract

**Objective:**

The effect of zeolite on Ca plasma concentration, reproductive parameters, body condition score (BCS), and colostrum properties was evaluated in Holstein dairy cows during the postpartum period.

**Materials and Methods:**

Sixty pregnant cows were allocated to three experimental groups, including 1) a control (CON) diet; 2) a CON diet + 0.75% dry matter (DM) zeolite (100 gm/day/cow); and 3) a CON diet + 1.5% DM zeolite (200 gm/day/cow). Experimental diets were fed to cows during the last 4 weeks of pregnancy. Blood sample evaluation for Ca concentration was done. The prevalence of hypocalcemia and reproductive parameters, including BCS and colostrum properties, was also measured.

**Results:**

Total Ca and ionized calcium (Ca^++^) concentrations in plasma at 6 and 12 h after calving were higher in zeolite-consuming cows than those in CON s (*p* < 0.01). Mean frequencies for severe and subclinical hypocalcemia in zeolite-consuming cows were 11.64% and 19.36% lower than those in the CON group, respectively (*p* < 0.01). The mean pregnancy efficiency of these cows was also 12.94% higher than the CON (*p* < 0.01). The mean BCS (*p* ≤ 0.05) and colostrum quality (*p* < 0.01) of zeolite-consuming cows were at maximum, but their mean colostrum yield was lower than that of the CON group (*p* < 0.01).

**Conclusion:**

Zeolite dietary supplementation at levels of 0.75% and 1.5% DM of the preparturient diet is recommended for better CON of hypocalcemia, improved BCS and colostrum quality, and better prevention of postpartum reproductive disorders in dairy cows.

## Introduction

The transition period is an important stage at the end of a dairy cow’s pregnancy. During this period, fresh cows will face many metabolic and reproductive challenges [1,2]. Hypocalcemia, or a dropping blood calcium concentration at calving time, is one of the most important abnormalities and is the cause of some diseases. Suppose cows do not have a proper nutrition regime during the transition period or are exposed to various stresses. In that case, they will suffer from many disorders that result in decreased productive and reproductive performance [3,4]. Various methods have been used to solve the problem and improve the health of fresh cows [1–5]. The most important solution is restricted calcium availability, and the second solution is to make the cation-anion difference of the diet dietary cation–anion difference (DCAD) negative [5–7]. Decreasing the DCAD value to a level of—100 milliequivalents per kilogram (meq/kg) of dry matter (DM) induces mild acidosis and the release of parathyroid hormone at the time of delivery [8,9], which finally increases the amount of calcium absorption from the bones and will prevent milk fever and hypocalcemia [2,3]. To reduce the availability of dietary calcium, restricted rations during the transition period based on National Research Council (NRC) recommendations are used to control (CON) hypocalcemia [10]. Still, practically, this method will cause some damage to the cows. Calcium homeostasis is a crucial factor during the transition period [11,12], and low-quality diets due to Ca restriction are associated with various negative effects on future cow performances [13,14]. Using natural calcium binders in feed to regulate the plasma Ca concentration around parturition seems like a practical and innovative solution [15,16–25]. Natural clinoptilolite and synthetic zeolites (Zeolite-A) have improved animal health and performance [17,18]. They were also used to absorb ammonia, heavy metal toxicities, and poisoning materials [19].

Clinoptilolite is a crystalline form with microporous aluminosilicate skeletons of alkaline earth cations for binding to divalent cations, fungal toxins, and harmful gases, as well as trapping the excess products [16,17]. Ion-exchange potential and the ability to reversibly lose and gain water are other unique properties of clinoptilolites [18,19]. Dietary restriction of calcium leads to a decrease in plasma calcium concentration and stimulation of the release of bone calcium stores [20,21]. Until now, many research studies have focused on applying clinoptilolite and zeolite to increase performance and improve the health of dairy cows. Katsoulos et al. [20] reported a significant reduction in postpartum milk fever and ketosis cases when they fed cows 2.5 gm clinoptilolite per 100 gm diet during the last weeks of the transition period. Cows fed with clinoptilolite in a long-term feeding program showed fewer cases of clinical ketosis and a higher total milk yield during the first month of postpartum. Also, concentrations of glucose, ketone bodies, blood urea nitrogen, and total protein in serum, as well as liver function, did not show any adverse effects of clinoptilolite supplementation. Increasing antibody production and immune response in dairy cows and calves vaccinated against *Escherichia coli* were found after daily intake of clinoptilolite [15,22–24]. Sinchi et al. [25] showed that dietary feeding of clinoptilolite reduces uterine polymorphonuclear (PMN) leukocytes and decreases open days in multiparous lactating dairy cows under a tropical mountain pasture-based management system. Their results confirmed a decreased frequency of cows with subclinical endometritis and a shortened calving interval in dairy cows.

Dietary supplementation with clinoptilolites may improve colostrum quality in fresh cows without adversely affecting performance, health, or serum biochemical parameters. In confirmation of this issue, Stojić et al. [18] reported that 150 gm per day oral clinoptilolite supplementation increases the IgG concentration in colostrum significantly without adverse effects on energy uptake or nutrient metabolism in primiparous dairy cows during the prepartum stage. Also, increased intestinal adsorption of immunoglobulins in the intestine with reduced incidence of diarrhea was observed in dietary clinoptilolite supplementation of dairy products [15,17].

Dietary zeolite-A was used to investigate immune functions due to changes in mineral availability (e.g., Ca, Mg, and P). Decreased mineral availability resulted in lower gene expression of neutrophil inflammatory mediators, suggesting that zeolite-A may CON inflammation during the prepartum period [26]. Regarding the productive and reproductive improvement of cows, zeolite has also shown its effectiveness. In this regard, Marin et al. [21] showed in a comprehensive study that dietary feeding of zeolite (150 gm per day per cow) has led to a decrease in the heavy metal content of milk, an increase in the concentration of unsaturated acids (oleic and linoleic), and immunoglobulins in colostrum, but a decrease in the cases of diarrheal disease. Also, zeolite supplementation during the prepartum period has led to a significant increase in serum Ca concentration both in the pre-calving and post-calving periods and fewer milk fever cases. Of course, in some reports, the effect of zeolite on serum mineral concentration has not been confirmed. Abdelrahman et al. [27] reported that after adding zeolite to the diet of growing lambs, serum, and rumen fluid mineral concentrations (P, Ca, and Mg) showed no significant differences. Conclusively, dietary zeolite supplementation at 2% improves performance and P digestibility, which may result in a lower P content in lamb’s waste and consequently reduced environmental pollution. It has been shown that dairy performance and ruminal characteristics were negatively affected when daily zeolite intake exceeded 400 gm per day. Therefore, as a key point, the exact level of zeolite intake has a significant impact on the response of lactating cows [17].

Almost no published data were found on the impact of clinoptilolite or zeolite on the body condition score (BCS) of cows, and there is also little information on reproduction parameters and colostrum characteristics. We know that adding clinoptilolite or zeolite to cow rations causes various beneficial results, but few findings have been reported on their effectiveness during the transition period. Furthermore, the dietary intake level of zeolite remains a knowledge gap that needs to be clarified. Therefore, the objective of the current experiment was to study the effect of different levels of dietary zeolite supplementation in the prenatal period on postpartum reproductive parameters, colostrum production, and BCS in fresh Holstein dairy cows.

## Materials and Methods 

### Ethical approval 

All procedures used on the animals in this study were approved by the Islamic Azad University Institutional Animal Care and Use Committee (reference number: IAUEC 2021/1400-265-33-11).

### Cows, housing and experimental diets 

Our experiment was performed on a commercial dairy farm (Fashafoyeh Agriculture and Industry Co., Pakdasht, Tehran, Iran) from June to September 2020. The mean initial BCS of the cows was 3.50 ± 0.25, and the average milk yield was 40 ± 2.20 kg/day. The thermal humidity index during the experiment was in the range of 71 to 83. Cows were housed individually after calving in a calving stall for 48 h. They were moved to a post-calving pen and fed a basal experimental diet in a total mixed ration twice daily at regular intervals. The experimental diet was adjusted according to NRC recommendations [10]. Ingredients and chemical compositions of experimental diets are shown in Tables 1 and 2.

Four weeks (28 days) before the expected calving date, the cows were moved to a close-up group and fed a zeolite diet with a DCAD of +100 mEq/kg DM. Sixty multiparous preparturient nonlactating Holstein--Friesian cows with a mean body weight (BW) of 754 ± 40 kg and a mean parity of 3.00 ± 0.50 were randomly assigned to experimental groups (three isoenergetic diets) based on previous 305-day milk yield, BW, BCS, and number of parities. Experimental groups were included: (1) CON diet without the zeolite supply CON, Ca 0.44%, DCAD + 100 mEq/kg; (2) CON diet + 0.75% DM. Zeolite supply (100 gm/day/cow), Ca 0.44%, DCAD + 100 mEq/kg (Z-100); (3) CON diet + 1.50% DM Zeolite supply (200 gm/day/cow), Ca 0.44%, DCAD +100 mEq/kg (Z-200). The zeolite supply (type A) used in this research was made by Iran Chemical Industries Company (nAl_2_O_3_.mSiO_2_.xH_2_O; two-layer and eight-sided; zeolite/Ca-binding ratio of 6:2; approved code: 2004-11-03 IZA) for use in animal nutrition. All cows were fed after calving with a normal postpartum diet.

### Blood and colostrum analyses 

After calving at hours 12 and 24 of postpartum (3 h after feeding), evacuated tubes containing heparin were used for blood samples from the coccygeal vessels. Samples were centrifuged at 3,000 × gm for 15 min, subsequently. The photometric method with specific kits (Pars Azmoon Chemical Company, Tehran, Iran, TS.M.91.13.4) was employed to measure total plasma Ca concentration according to the instructions provided by the manufacturer using an analyzer (Roche Hitachi-911, Chemistry Analyzer Company). The concentration of plasma-ionized Ca was measured using the ion-selective electrode method with an electrolyte analyzer (AC-9800, Audicom Company). The cows were milked within 30 min after calving for colostrum specimens (2 samples). One specimen (250 ml) was used to measure the weight and quantity, and the second sample was used to measure the colostrum quality via a Brix refractometer. A Brix refractometer (Model LH-Y12, Atago Co., Ltd., Tokyo, Japan) was used to determine the concentration of IgG (Brix%). IgG concentrations in colostrum are highly correlated with Brix values determined by a radial immunodiffusion assay, as described by Bielmann et al. [28].

**Table 1.** Feed ingredients of the experimental diets before and after parturition (% of DM).

Ingredients	Prepartum period			Postpartum period
	CON	Z-100	Z-200	
Alfalfa	16.11	15.97	15.86	17.20
Corn silage	31.11	30.87	30.63	28.70
Wheat straw	6.37	6.34	6.28	2.20
Wheat bran	3.94	3.93	3.88	-
Barley	11.82	11.73	11.65	13.50
Corn	11.96	11.86	11.78	15.80
Soybean meal 44%	10.94	10.86	10.77	11.93
Canola meal	3.57	3.54	3.52	3.95
Corn gluten meal 60%	1.88	1.87	1.85	1.95
Propylene glycol	0.98	0.97	0.97	1.10
Vitamin-mineral premix^A ^	0.98	0.97	0.97	1.20
Zeolite^B^	-	0.75	1.50	-
Calcium carbonate	-	-	-	0.60
Magnesium oxide	0.34	0.34	0.34	0.30
Dicalcium phosphate	-	-	-	0.22
Sodium bicarbonate	-	-	-	1.20
Sodium chloride	-	-	-	0.15
Total	100	100	100	100

Diets: CON (control); Z-100 (zeolite 100 gm/day/cow); Z-200 (zeolite 200 gm/day/cow). ^A^Each kilogram vitamin-mineral premix contained: 19 gm Mg, 12 gm Fe, 10 gm Mn, 13 gm ZN, 300 mg Cu, 100 mg Co, 30 mg Se, 100 mg I, 5 million IU vitamin A, 1 million IU vitamin D3, and 30 mg vitamin E. ^B^Zeolite, produced by Iranian Chemical Co. (general formula: nAl_2_O_3_.mSiO_2_.xH_2_O), a two-layer and eight-sided class of clinoptilolite with zeolite/Ca binding ratio of 6:2. DM = 90%, DCAD = 3,400 milliequivalents per kilogram of DM.

**Table 2.** Chemical composition of the experimental diets before and after parturition (% of DM)^A^.

Nutrients	Prepartum period			Postpartum period
	CON	Z-100	Z-200	
CP	16.24	16.24	16.24	16.54
NE_L_^2^ (Mcal/kg)	1.58	1.58	1.58	1.68
NDF^3^	35.50	35.50	35.50	31.74
ADF^4^	21.00	21.00	21.00	19.22
NFC^5^	38.70	38.33	38.33	40.54
EE^6^	2.65	2.70	2.70	3.31
Ash	6.95	6.95	6.95	7.31
Ca	0.44	0.44	0.44	0.75
P	0.35	0.35	0.35	0.39
Mg	0.44	0.44	0.44	0.40
Cl	0.16	0.16	0.16	0.23
K	1.26	1.26	1.26	1.31
Na	0.05	0.05	0.05	0.38
S	0.22	0.22	0.22	0.25
DCAD^7^ mEq/kg^8^ DM	+100	+100	+100	+300

Diets: CON (control); Z-100(zeolite 100 gm/day/cow); Z-200 (zeolite 200 gm/day/cow). ^A^Determined on samples pooled by week (*n =* 8). ^B^NE_L_: net energy for lactation (based on tabular values of NRC, 2001), ^C^NDF: neutral detergent fiber, ^D^ADF: acid detergent fiber, ^E^NFC: non-fiber carbohydrate (100—NDF neutral detergent insoluble CP) + (CP + ash + fat), ^F^EE: ether extract, ^G^DCAD: dietary cation–anion difference [(Na^+^ + K^+^) – (Cl^–^ + S^–2^)], ^A^mEq/kg: milliequivalents per kilogram of diet.

### Feed analyses

All feed samples were ground through a 1 mm screen using a Wiley mill before the analyses (Arthur Thomas Co., Philadelphia, PA). An oven-drying method was used for determining the DM at 65°C until a constant weight was reached; method 930.15. Samples were analyzed by a Kjeldahl titration instrument (Kjeltec 1030 Auto Analyzer, Tecator, Höganäs, Sweden) for crude protein (CP) analysis (*N* × 6.25), according to AOAC [29]; method 920.53. Also, ether extract (EE) and ash contents of feed samples were determined according to AOAC [29] methods 920.39 and 941.12. neutral detergent fiber (NDF) and acid detergent fiber (ADF) were measured using heat-stable amylase (100 l/0.5 gm of sample) and sodium sulfite [30].

### Calculation and data correction 

The frequency of each case (number of abnormalities) was obtained to calculate the prevalence of reproductive disorders as a corrected percentage for different groups. Before analysis, the normal distribution of the data were tested. All discontinuous or numerical variables were corrected using a standard statistical formula (ArcSin √X).

### Statistical analysis

A completely randomized statistical design completely randomized design was used to analyze the data using SAS 9.2 [31]. Continuous data were analyzed by two-way variance analysis using the MIXED Procedure (Proc Mixed) with repeated observations. For discontinuous data (e.g., frequencies of reproduction disorders), nonparametric analysis was used. The Shapiro–Wilk test was used to ensure the normal data distribution before analysis. The mean differences between treatments were compared using Duncan’s multiple range test at *p* ≤ 0.01.

## Results

### Plasma Ca concentration 

The effects of different levels of zeolite on the Plasma Ca concentration of cows from calving time until 24 h after parturition are presented in Table 3. At the calving time, the total Plasma Ca concentration of the CON group was at its maximum (7.43 mg/dl), and that of the Z-100 and Z-200 groups was at its minimum (7.35 and 7.32 mg/dl, respectively) (*p* < 0.01). A similar trend for Ca^++^ plasma concentration was observed (*p* < 0.01); however, the ratio of Ca^++^/Ca was insignificant between the groups. But at 12 h after parturition, the situation has completely changed, so the highest values of total and ionized plasma Ca concentration were for zeolite-consuming groups compared to CON (8.53 and 8.40 *vs*. 8.38 mg/dl for total Ca and 4.10 and 4.11 *vs*. 4.02 mg/dl for Ca^++^) (*p* < 0.01). With minor differences, the same trend was observed 24 h after parturition (*p* < 0.01). Therefore, the positive effect of zeolite-containing diets on CON ling Plasma Ca concentration in the cows was confirmed.

### Frequency of hypocalcemia and reproductive disorders 

The effects of different levels of zeolite on the frequency of hypocalcemia (severe and subclinical) and other post-calving reproductive disorders are given in Table 4. The frequency of severe hypocalcemia as a corrected percentage for the CON, Z-100, and Z-200 groups was 40.45%, 36.27%, and 35.21%, respectively. So, a significant difference was observed between the groups (*p* < 0.01). The frequency change trend of severe hypocalcemia from CON to Z-200 was downward. Similarly, the same trend was observed for most reproductive traits except for pregnancy efficiency (*p* < 0.01). Other characteristics such as calving difficulty, abomasal displacement, and culled cows in zeolite-consuming groups were not observed in any cases compared to CON (*p* < 0.01). The use of zeolite at both levels caused a significant improvement in the mean percentage of retained placenta and endometritis. The pregnancy efficiency was maximum for zeolite-consuming groups (*p* < 0.01). This is an important indicator of fertility in zeolite-consuming groups compared to the CON group.

### BCS and colostrum characteristics 

The effects of different levels of zeolite on BCS and colostrum characteristics are shown in Table 5. The BCS of cows in the 7 days before parturition was insignificant. However, the BCS score and BCS reduction score on the 30th day after parturition for the CON-group were lower and higher than the zeolite-consuming groups, respectively. The BCS score of the zeolite-consuming groups (2.93 and 2.98) against the CON (2.87) was significantly higher (*p* < 0.05). Also, the BCS reduction score for them was 0.45 and 0.43, which was significantly (*p* < 0.05) lower than CON‘s 0.55. The daily colostrum production in the CON group was significantly higher than in the zeolite-consuming groups (6.62 *vs.* 652 and 6.36 kg/d). But conversely, the quality of colostrum produced by zeolite-consuming groups was significantly (*p* < 0.01) higher than that of the CON group (23.8% and 23.64 *vs.* 22.38%).

**Table 3.** Effect of zeolite supplementation on Plasma Ca concentration (mg/dl).

Plasma Ca concentration (mg/dl)	Diets			SEM	*p*-value		
	CON	Z-100	Z-200		Trt	Time	Trt × Time
At parturition							
Ca^A^	7.43^a^	7.35^b^	7.32^b^	0.037	0.011	0.033	0.035
Ca^++B^	3.53^a^	3.49^ab^	3.44^b^	0.053	0.002	0.015	0.032
Ca^++^/Ca^C^	0.47	0.48	0.47	0.013	0.233	0.255	0.465
12 h postpartum							
Ca	8.38^b^	8.40^b^	8.53^a^	0.058	0.002	0.001	0.001
Ca^++^	4.02^b^	4.11^a^	4.10^a^	0.054	0.001	0.001	0.001
Ca^++^/Ca	0.48	0.48	0.48	0.010	0.333	0.423	0.455
24 h postpartum							
Ca	8.71^b^	8.79^ab^	8.88^a^	0.061	0.015	0.027	0.044
Ca^++^	4.03^b^	4.16^a^	4.21^a^	0.056	0.005	0.039	0.038
Ca^++^/Ca	0.49	0.47	0.47	0.011	0.689	0.825	0.844

^abc^Different superscript letters above values indicate a significant difference of means (*p* ≤ 0.01). Diets: CON (control); Z-100(zeolite 100 gm/day/cow); Z-200 (zeolite 200 gm/day/cow). SEM: Standard Error of Mean. ^A^Ca: Total plasma Ca concentration; ^B^Ca^++^: Ionized plasma Ca concentration; ^C^Ca^++^/Ca: Ratio of Ca^++^ to Ca.

## Discussion

### Plasma calcium concentration 

The changes in plasma calcium concentration at parturition, 12 h after parturition, and 24 h after parturition are shown in Table 3. Total and ionized Plasma Ca concentrations of the CON group at parturition were higher than those of the Z-100 and Z-200 groups. Still, over time, at 12 and 24 h after parturition, the Plasma Ca concentration of the zeolite-consuming groups was higher than that of the CON group. Therefore, from departure until hours after that, the situation changed completely. In all experimental groups, a normal range of Plasma Ca concentration was observed immediately after parturition. This situation was consistent with most reports [3,14,32]. A review of the reports indicates that an increase in the Plasma Ca concentration was observed following the Ca bolus prescription [3,12] because the plasma Ca concentration directly depends on dietary Ca level and ruminal Ca concentration [2,14]. However, in this experiment, there was no Ca bolus administration or any other dietary Ca supplement. Therefore, the only factor affecting the regulation of plasma Ca concentration is physiological and hormonal mechanisms due to the induction effect caused by zeolite. In line with our findings, dietary zeolite supplementation during the last week of the pre-calving period significantly increased blood serum calcium concentration [2,14]. Conclusively, available reports have shown that feeding clinoptilolite or zeolite in the prepartum ration of dairy cows can reduce the frequency of hypocalcemia and prevent milk fever because zeolite can stabilize calcium in the intestinal tract and make it unabsorbable, which stimulates the physiological and hormonal mechanisms involved in increasing the level of blood Ca concentration [2,8,18,21,32].

It has been shown that Plasma Ca concentration will increase by 0.1 mmol/l after oral Ca prescription per dose [2,3,12]. As the results of Table 3 confirmed, the level of plasma Ca concentration for zeolite-consuming groups was always 0.1 mmol/l higher than the CON group 12 and 24 h after parturition due to the effect caused by using zeolite. The results of several studies confirmed that using anionic materials in the prepartum period as a routine strategy cannot prevent the occurrence of hypocalcemia on the first day of parturition completely. In this situation, it is necessary to use an auxiliary supplement such as an oral Ca bolus for cows [9,13,32,33]. Some inconsistencies can be seen in the results of different reports. Still, it should be remembered that critical thresholds for the prevalence of clinical and subclinical symptoms of hypocalcemia have not yet been accurately defined [4,9,13]. The report of Oetzel [13] and further investigations indicate that the critical threshold of Plasma Ca concentration at parturition time is less than 7.3 mg/dl (equal to 1.825 mmol/l), which leads to the onset of clinical symptoms. The critical threshold for subclinical hypocalcemia is less than 8.5 mg/dl (equal to 1.2 mmol/l). The results of the current study confirmed the mentioned findings. Also, it was concluded that the administration of 100 to 200 gm/day/cow of zeolite during the last 2 to 4 weeks of pregnancy caused a significant increase in serum Ca concentration on the day of calving until 24 h after that.

### Frequency of hypocalcemia and reproductive disorders

The results of Tables 3 and 4 indicated that, in all experimental groups, several hypocalcemic cows were observed. Hypocalcemic cows were less common among groups after parturition due to increased Plasma Ca concentration. So, the highest plasma Ca concentrations belonged to zeolite-consuming groups. Some reports suggest that if the critical threshold of Plasma Ca concentration is less than 8.5 mg/dl, the common measures will not prevent subclinical hypocalcemia [1,6,13]. Our findings indicated that zeolite-consuming cows always had a higher Plasma Ca concentration than the mentioned critical threshold. Therefore, the efficiency of feeding zeolite was confirmed compared to the CON diet. Hypocalcemia is the main reason for milk fever, an important disease in dairy farms, which involves abundant losses to the dairy industry. It intensifies due to the cow’s inability to maintain the optimal Serum Ca concentration following parturition. Hypocalcemia manifests in clinical or subclinical form, depending on Ca serum concentration. If the number of diagnosed cases is greater than 10%, it is necessary to employ a specific CON program to prevent disorders [2,4,9,21]. According to available reports, dietary clinoptilolite supplementation during the prepartum period could be a preventive action for hypocalcemia, parturient paresis, and milk fever [9,18,20]. A minimum efficiency of 58% for dietary zeolite supplementation was established to prevent hypocalcemia on the day of calving [21]. Therefore, a novel nutritional strategy to prevent milk fever is based on the effect of zeolite on Blood Ca concentration. According to our findings, we can conclude that dietary zeolite supplementation could be part of this preventive strategy.

All disorders in this study responded positively to dietary zeolite supplementation, and their frequency was lower than CON (Table 4). Moreover, the percentage of pregnancy efficiency (pregnancy percentage on the 150th day after parturition) was significantly higher than CON for zeolite-consuming groups. The beneficial effect of zeolite supplementation in the present study is consistent with reports that imply improved productive and reproductive traits [22,23,25,14] and pregnancy performance due to improved digestion or metabolic status in cows. The range of hypocalcemia prevalence stated in different reports ranged from a minimum of 33% in the study of Jahani-Moghadam et al. [3] to a maximum of 58.6% in the study of Amanlou et al. [33], who reported higher hypocalcemia occurrence for the prepartum cows fed DCAD diets with a Ca serum concentration of less than 2.125 mmol/l immediately after parturition. A large field survey showed that, at less than 1 day-in-milk, hypocalcemia symptoms were observed in 64% of cows in the third lactation period fed an anionic supplement [34]. These findings indicate that a diet supplemented by anionic salts can only CON part of the occurrence of hypocalcemia. Still, due to the influence of other factors, more investigations are needed. According to Jahani-Moghadam et al. [3], the lowest frequency of hypocalcemia in the first 48 h was observed in the Ca bolus-supplemented groups. Therefore, CON of Plasma Ca concentration by an efficient method may be placed as a prophylactic action for all cows before calving.

The percentage of retained placenta in the zeolite-consuming group was also significantly lower than CON (26.56% *vs.* 31.10%). Although the acceptable criteria for retained placenta in different reports were stated as 20% [13,35], Coordinately, the results of the present study indicate that dietary zeolite supplementation for the experimental groups had a greater preventive effect against the incidence of retained placenta than in normal conditions. In this connection, some findings indicated that the occurrence of retained placenta in cows consuming negative DCAD diets was lower than in normal diets [5,9]. Less frequent retained placenta in our study is probably due to higher postpartum Plasma Ca concentrations in zeolite-consuming cows. Similarly, the frequency of endometritis in the cows of zeolite-consuming groups was lower than CON (30% *vs.* 32.21%), which confirms the positive effect of zeolite supplementation. Also, there were no calving difficulty, abomasal displacement, or removal of cows from the herd in the zeolite-consuming groups. Also, other abnormalities in these cows were less frequent than CON. These findings are consistent with the reports that confirm the beneficial effect of zeolite supplementation in CON ling Plasma Ca concentration and improving reproductive parameters [16,20,21,25].

The daily addition of zeolite to the prepartum diet of multiparous lactating dairy cows significantly reduced the number of uterine PMN leukocytes (an index of uterine health), the frequency of cows with subclinical endometritis, and the calving to conception interval [25]. Clinoptilolite can improve uterine health and subsequent conception rates through two mechanisms, including: 1) modifying ruminal physiology and improving the cow’s metabolic status [17] and 2) improving the cow’s immune system [15,18,25,36].

Uterus bacteria colonization occurs during the calving process, and many of them are pathogenic and connect to the endometrium and penetrate deeper layers, causing infections and infertility [11,37]. Accelerating the resumption of cyclic ovarian activity after parturition is vital to achieving maximum dairy herd efficiency [2,11,19]. In this regard, according to Sinchi et al. [25], larger follicles were determined in clinoptilolite-consuming cows when ultrasonographic monitoring was performed. The results are consistent with the improvement in other reproductive indices, such as first corpus luteum occurrence, first conception rate, frequency of cows with a corpus luteum at day 30 after calving, and significant improvement in open days. Also, dietary clinoptilolite supplementation of dairy heifers with 200 gm/day/cow reduced by 6 days the interval to the first post-calving estrous [23]. The findings of our research are consistent with the results of the mentioned reports.

**Table 4.** Effect of zeolite supplementation on postpartum prevalence of hypocalcemia and reproduction disorders.

Items (frequency^A^)	Diets			SEM	*p*-value
	CON	Z-100	Z-200		
Severe hypocalcemia	40.45	36.27	35.21	3.33	0.000
Subclinical hypocalcemia	34.19	28.12	27.02	2.12	0.000
Calving difficulty	18.44	0	0	0.16	0.011
Retained placenta	31.10	26.56	26.56	2.11	0.002
Endometritis	32.21	30.00	30.00	1.15	0.014
Abomasal displacement	17.55	0	0	1.74	0.002
Culled Cows	12.92	0	0	2.35	0.014
Pregnancy efficiency^3^	53.73	60.00	63.43	2.18	0.000

^abc^Different superscript letters above values indicate a significant difference of means (*p* ≤ 0.01). ^A^Frequencies were reported based on corrected percentage for cows with signs of disorder calculated by ARCSin√X formula. Diets: CON (control); Z-100(zeolite 100 gm/day/cow); Z-200 (zeolite 200 gm/day/cow). ^B^Pregnancy% in 150th day after parturition. SEM: Standard Error of Mean.

**Table 5.** Effect of zeolite supplementation on BCS and colostrum characteristics.

Items	Diets			SEM	*p*-value		
	CON	Z-100	Z-200		Trt	Time	Trt × Time
BCS^A^ 7 days before parturition	3.42	3.38	3.41	0.129	0.343	0.730	0.855
BCS 30 days after parturition	2.87^b^	2.93^a^	2.98^a^	0.044	0.022	0.002	0.033
BCS reduction score	0.55^a^	0.45^b^	0.43^b^	0.033	0.021	0.024	0.031
Colostrum production (kg/day)	6.62^a^	6.52^ab^	6.36^b^	0.123	0.005	0.046	0.055
Colostrum quality (Brix %)^B^	22.38^b^	23.64^ab^	23.80^a^	0.303	0.004	0.535	0.431

^abc^Different superscript letters above values indicate a significant difference of means (*p* ≤ 0.01). Diets: CON (control); Z-100(zeolite 100 gm/day/cow); Z-200 (zeolite 200 gm/day/cow). ^A^BCS: Body condition score. ^B^Determining the quality of colostrum was done based on the standard method of colostrum quality measurement based on Brix% with Brix refractometer.

SEM: Standard Error of Mean.

### BCS and colostrum characteristics 

The BCS of the cows 7 days before parturition for all groups was not different, but on the 30th day after parturition, the BCS between groups was significantly different (Table 5). The highest BCS was for Z-200 (2.98) and then for Z-100 (2.93), against the lowest value (2.87) for CON. Reversely, the highest BCS reduction score was observed in the CON group (0.55), which was significantly different from the Z-100 (0.45) and Z-200 (0.43) groups. These findings confirm the beneficial effect of zeolite on body energy balance and the cow’s metabolic status, which is reflected in improved BCS [38]. BCS is an easy-to-use index that expresses the cow’s body condition at different stages of breeding [38–40]. A different method of the BCS scale can be used to evaluate cows [38,40]. There is a strong correlation between the BCS of a cow and her future reproductive efficiency. Also, BCS is the greatest factor influencing the performance of cows at parturition time [39]. So, the frequency of cows in the open phase, calving interval, and calf vigor rate at calving time are all closely related to the BCS of cows at all times [38,40]. It is difficult to establish suitable conditions for cows after parturition, and more energy and nutrients are needed after parturition. Therefore, the best tool to manage dairy cows is monitoring the BCS during key times before and after calving [38,39].

In line with the results of this study, Karatzia et al. [23] reported that dietary clinoptilolite supplementation with 200 gm/day/cow increased BCS, serum glucose, and acetoacetate concentrations and decreased serum ketone body concentrations without adverse effects on milk production [23]. No other published reports were found about the impact of clinoptilolite or zeolite on BCS to compare with our results.

It is well shown that a suitable energy balance results in an acceptable BCS, and an appropriate postpartum BCS influences reproductive efficiency positively [25,38]. BCS-related factors play a crucial role in the cow’s productivity, finally affecting the frequency of vigor calves produced [38–40]. According to research findings, the appropriate BCS is about three around parturition. The cows with lower BCS will not have enough energy to support milk production in the early stages of production after parturition. Cows with very high or low scores are prone to metabolic and infectious diseases as well as reproductive disorders [35,37]. Also, cows with abnormal BSC show a sharp and rapid decrease in their weight at the beginning of calving, leading to a decrease in their fertility and milk production [38,39]. As a key result, monitoring BCS could increase the herd’s profitability.

Colostrum production (kg/d) and colostrum quality (Brix%) were other characteristics that were compared among the groups. According to the results given in Table 5, the trend of these two characteristics was generally different. The highest colostrum production value was for the CON group (6.62 kg/day) against the Z-100 (6.52 kg/day) and Z-200 (6.36 kg/day) groups. Reversely, the lowest colostrum quality, with a percentage of 22.38 was for CON, against 23.64% and 23.80% for Z-100 and Z-200, respectively. These findings show that despite the quantitative superiority of the CON group in terms of colostrum production, qualitatively, due to the positive effect of zeolite, this group had the lowest value among the groups. Also, these findings are in agreement with the results that confirm the beneficial effect of zeolite supplementation on the qualitative characteristics of milk and colostrum production in cows [18,22,36]. Intestinal pinocytosis mechanisms mediate colostrum IgG absorption from intestinal cells, which works only 24 h after birth. The high mineral content of colostrum can accelerate the secretion of pinocytosis through intestinal epithelial cells. Consequently, high colostrum immunoglobulin content is connected to acceptable health status and less frequent diarrhea and mortality in newborn calves [21]. There was also a strong positive relationship observed between total solids content, proteins, and sugar percentage of colostrum and immunoglobulin concentration, which was reflected in colostrum quality stated as the Brix index [24,36].

Improving the performance, health, and safety of dairy cows, as well as protection against mycotoxin intoxication, are among the reasons that have spread the use of natural and synthetic zeolites in recent years [16–18]. Mohri et al. [22] investigated the impacts of short-term clinoptilolite supplementation in colostrum and milk on some serum mineral concentrations in newborn dairy calves. Their report indicated that clinoptilolite supplementation could promote serum mineral concentrations with better hemopoiesis and prevent pathologic or physiologic disorders in clinoptilolite-consuming calves during the first weeks after birth. Clinoptilolite and zeolite supplementation increase available serum Ca concentrations. This issue is important to meet the higher need for Ca in growing calves. Therefore, a significant increase can be expected in the serum concentration of minerals during a short-term feeding of clinoptilolite in newborn calves without any adverse effects.

A serum and colostrum increase of specific antibody titers against *E. coli* was observed after administration of 200 gm/day of clinoptilolite for 70 days in lactating cows [15]. Also, 50% greater IgG levels at 24 and 48 h after birth were reported after colostrum supplementation (3 l of colostrum containing 5 gm/l of clinoptilolite) of neonatal calves with clinoptilolite [36]. According to the report of Marc et al. [24], serum protein composition in newborn calves can be changed after clinoptilolite consumption. Their results indicated that γ-globulins, β-globulin, and total protein concentrations in zeolite-consuming calves at 30 h after birth were higher than CON (42.11% and 28.48% *vs.* 18.52%). These changes, combined with a less significant albumin/globulin ratio in zeolite-consuming calves (29.35%) than in the CON group at the same age, indicated a significant increase in the globulin fraction in experimental calves. As a result, clinoptilolite improved passive transfer in neonatal calves, but it will be more effective if added at a lower dose (0.5% in colostrum) than at a higher dose. The exact mechanism of clinoptilolite and zeolite enhancement of immune activity in animals has not yet been elucidated, but a large body of evidence clearly supports the immune-stimulating effect of clinoptilolite and zeolite. It seems that prepartum dietary zeolite administration in dairy cows improves postpartum colostrum quality, which consequently affects the health and passive immunity of new-born calves. In conclusion, prepartum dietary supplementation of clinoptilolite or zeolite in dairy cows may be employed as a routine program to improve health, BCS, colostrum quality, and reproductive efficiency in the postpartum period, but it should be noted that considering necessary conditions such as consumption level, the exact duration of use, type purity, and physico-chemical properties of the compound, as well as dietary mixing method and diet specifications, is very effective in producing favorable results.

## Conclusion

According to our findings, supplementing the prepartum diets with different levels of zeolite (100 and 200 gm/day/cow) prevented hypocalcemia through parturition and after that, which resulted in higher total and ionized plasma Ca concentration after calving in zeolite-consuming cows than in the CON group. Further, dietary zeolite supplementation improved reproductive traits as well as BCS and colostrum quality. Less frequent severe and subclinical hypocalcemia and other reproductive disorders were observed in zeolite-consuming cows than in the CON group. BCS and colostrum quality were higher for zeolite-consuming cows than the CON group. Therefore, feeding zeolite to cows before parturition could be suggested; however, due to a lack of sufficient information in this field, more extensive studies are needed.

## List of Abbreviations

BCS, body condition score; BW, body weight; Ca, calcium; d, day; DM, dry matter; CON, control; DCAD, dietary cation-anion difference; kg, kilogram; mmol, milimol; NRC, national research council; NS, not significant; SE, standard error; SEM, standard error of mean; *p*-value, Probability value or index; vs., versus.
